# The use of individual and multilevel data in the development of a risk prediction model to predict patients’ likelihood of completing colorectal cancer screening

**DOI:** 10.1016/j.pmedr.2023.102366

**Published:** 2023-08-11

**Authors:** Amanda F. Petrik, Eric S. Johnson, Rajasekhara Mummadi, Matthew Slaughter, Gloria D. Coronado, Sunny C. Lin, Lucy Savitz, Neal Wallace

**Affiliations:** a Kaiser Permanente Center for Health Research, Portland, OR, USA; b Northwest Permanente, Portland, OR, USA; c Oregon Health & Science University/Portland State University School of Public Health, Portland, OR, USA; d Washington University, St. Louis, MO, USA; e University of Pittsburgh, Pittsburgh, PA, USA

## Abstract

Promotion of colorectal cancer (CRC) screening can be expensive and unnecessary for many patients. The use of predictive analytics promises to help health systems target the right services to the right patients at the right time while improving population health. Multilevel data at the interpersonal, organizational, community, and policy levels, is rarely considered in clinical decision making but may be used to improve CRC screening risk prediction. We compared the effectiveness of a CRC screening risk prediction model that uses multilevel data with a more conventional model that uses only individual patient data.

We used a retrospective cohort to ascertain the one-year occurrence of CRC screening. The cohort was determined from a Health Maintenance Organization, in Oregon. Eligible patients were 50–75 years old, health plan members for at least one year before their birthday in 2018 and were due for screening. We created a risk model using logistic regression first with data available in the electronic health record (EHR), and then added multilevel data.

In a cohort of 59,249 patients, 36.1% completed CRC screening. The individual level model included 14 demographic, clinical and encounter based characteristics, had a bootstrap-corrected C-statistic of 0.722 and sufficient calibration. The multilevel model added 9 variables from clinical setting and community characteristics, and the bootstrap-corrected C-statistic remained the same with continued sufficient calibration.

The predictive power of the CRC screening model did not improve after adding multilevel data. Our findings suggest that multilevel data added no improvement to the prediction of the likelihood of CRC screening.

## 1 Background

Use of predictive analytics in healthcare has been steadily rising as health systems leaders are recognizing their value in targeting and optimizing care for patients at high risk of negative health outcomes (Miller, 2019). Predictive analytics is a way to maximize the utility of healthcare expenses through precision delivery of care in our resource-constrained healthcare climate (Bresnick, 2018). Understanding which patients most benefit from outreach can help care delivery systems prioritize spending on patients who need it most.

Risk prediction models can be used to identify populations at risk for disease or adverse events (Jeffery et al., 2019, Benuzillo et al., 2019). However, models have not always been built using a broad patient population or multi-level data (Jeffery et al., 2019, Moons et al., 2019, Parikh et al., 2019, Kent et al., 2018). Multilevel data can reveal influences that individual level data cannot. While individual characteristics may predict health behaviors such as cancer screening, emerging evidence is increasingly pointing to the outsized role of external factors such as interpersonal (friends, family, providers), organizational data (clinic setting and provider characteristics), community characteristics and other relevant publicly available data (census data) in influencing behaviors. (Zapka et al., 2012) The use of predictive analytics in combination with multi-level data could be one way to recognize group membership and individual characteristics simultaneously. Advancements in the application of predictive analytics in addressing health needs may include increasing access to more diverse sources of data.

To our knowledge, predictive analytics have not been applied to predict a patient’s likelihood of ***screening*** for colorectal cancer (CRC). CRC screening can save lives because precancerous polyps or early stage cancers are detected and removed (Safayeva and Bayramov, 2019). Yet, only 69% of age eligible adults in the general population were screened for CRC in 2020. (Richardson et al., 2022) This means that a staggering 21.7 million people are not up to date on their CRC screening (CDC, 2018). Patient, provider, and system level characteristics have been found to contribute to an individual’s failure to screen for CRC (Muthukrishnan et al., 2019, Yu et al., 2018, Weiss et al., 2013), suggesting that a multi-level risk prediction model might identify patients who are unlikely to screen for CRC.

This study sought to develop and validate a risk prediction model to predict patients’ likelihood of screening for CRC. First, we used individual-level data available from the electronic health record (EHR) to predict CRC screening and compared the performance of this model to a model where we added multilevel data obtained from US Census, American Community Survey, Department of Health, and locally available data.

## 2 Methods

### 2.1 Setting and patient population

The study was conducted at Kaiser Permanente Northwest (KPNW), an integrated health and financing system that provides health insurance coverage to about 606,000 members and dental insurance coverage to approximately 280,000 members in northwest Oregon and southwest Washington, in the United States. The study met the KPNW guidelines for protection of human subjects concerning safety and privacy (KPNW IRB #00000405, 1/6/21). The outcome of interest was CRC screening completion in 2019, based on HEDIS criteria, including fecal testing, FIT DNA, or colonoscopy in the year. (Centers for Disease Control and Prevention) All study procedures were reviewed and approved by the KPNW institutional review board; informed consent was waived as a data only study.

**Fig. 1:**
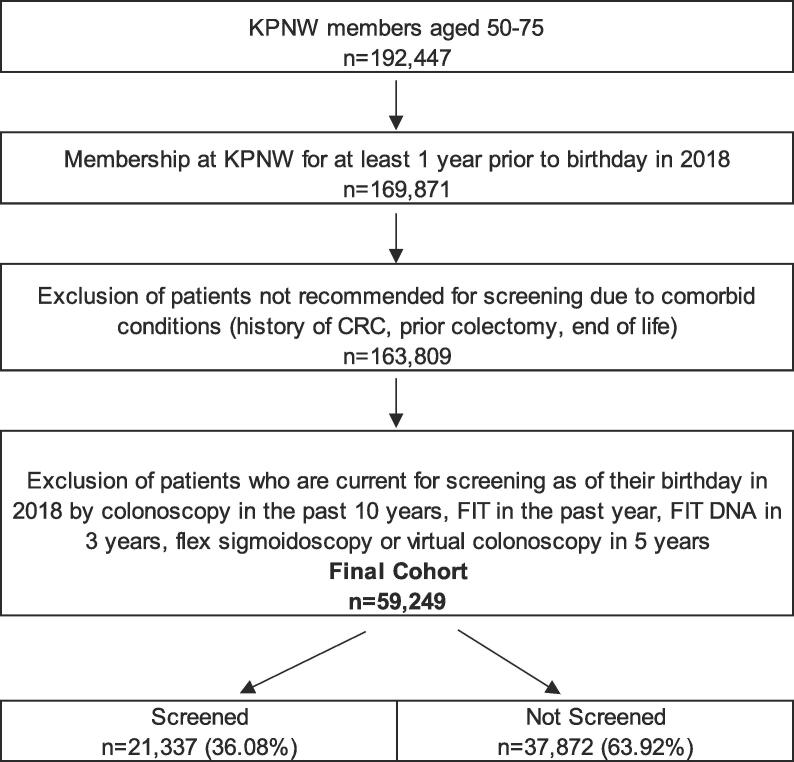
Eligible Patient Consert Diagram.

Eligible patients were identified through the EHR at KPNW (Fig. 1). Patients were determined eligible if on their birthday in 2018 if they were due for screening and aged 50–75 (n = 192,447). Eligible patients had to have at least 1 year of membership prior to their birthday (n = 169,871). Predictors were included from clinical encounters closest to but prior to their birthday in 2018. Outcomes were assessed for up to one year following their birthday. Patients were excluded if they were not recommended for screening due to comorbid conditions like a history of CRC, had a prior colectomy or if they were in end-of-life care (n = 163,809 remaining eligible patients) (Centers for Disease Control and Prevention, 2020). Patients were also excluded if they were current for screening by way of FIT in the past year, colonoscopy in the past 10 years, FIT DNA in the past 3 years, or flex sigmoidoscopy or virtual colonoscopy in 5 years (n = 104,617 (64%) excluded) (Centers for Disease Control and Prevention, 2023). Of the remaining 59,249 eligible patients, 36% were found to have been screened, which is the primary outcome for analysis.

### 2.2 Model development

The design of this study was guided by the Social Ecological Model (SEM) multilevel approach to colorectal cancer promotion, which suggests multiple bands of influence (individual, interpersonal, organizational, community, policy) (Zapka and Lemon, 2004). The selection of potential predictors was guided by literature on predictors of CRC screening, potential measures were identified by level of influence in the SEM framework. Available data included 21 individual level, 5 interpersonal level, 5 organizational level, 29 community level and 1 policy level variables. Individual level predictors included individual level data available in the EHR (demographic, encounter, enrollment, diagnosis, and procedure data). Interpersonal level predictors were also from the EHR (marital status, physician recommendation, provider characteristics). Organizational level predictors included administrative data (clinic characteristics). Community level predictors included Census and County level data (i.e., education by census block, median household income, neighborhood deprivation index, percent Hispanic ethnicity, rurality), and policy level predictors included State characteristics (CRC screening rates, Medicaid expansion, incentivized metrics). For EHR data, all predictors were identified in clinical records closest to but prior to the patient’s birthday in 2018. Predictors were defined and assessed in the same way for all participants. Predictor assessments were made without knowledge of the outcome data. Transparent reporting of a multivariable prediction model for individual prognosis or diagnosis (TRIPOD) reporting guidelines were used in development of this manuscript. (Collins et al., 2015).

Characteristics of the population of patients due for screening were assessed for variability in demographics, such as race, ethnicity, or insurance type. Some predictors were excluded prior to use due to unavailability (i.e., immigration status, income), missingness (i.e., education level, provider race/ethnicity match), or lack of distribution (i.e., having a primary care provider, or health literacy score). The Prediction Risk of Bias Assessment Tool (PROBAST) was used to reduce the risk of bias (ROB) by adhering to the 20 PROBAST principles (Wolff et al., 2019). Participants, predictors, outcomes, and analysis bias was assessed through questions to determine population characteristics disproportionate to the overall population, predictors with missingness in populations, and outcomes ratios based on the original population. Then observed screening rates for different groups identified in PROBAST were evaluated for differences (in % screened) from the overall eligible population. The sources of data were assessed for the ROB and the applicability to the question. The final predictors were assessed for missingness for all eligible patients in the model, and screening completion determined prior to analysis.

### 2.3 Model performance Assessment

Characteristics predicting the likelihood of screening were evaluated using a multivariate logistic regression model in SAS® System Software. The model assumes an absence of screening for patients who died or discontinued insurance coverage in the year of follow-up. For the first model, only available individual level data from the EHR and administrative datasets was used. The multilevel model incorporated the external multilevel data, and then tested the applicability of the final model to a subpopulation of patients who have been historically underserved by the medical system including African American or Black, Asian, Native American, Native Hawaiian, Pacific Islander (non-White).

For each model, a full model of patients with complete data for that model was fit. Then, guided by Harrell’s methods, a step-down method was used to manually remove the least predictive characteristics one covariate at a time to make the model parsimonious such that the final model retained at least 95% of the variation explained in the full model. (Heinze et al., 2018, Harrell et al., 1998).

For the final model, the observed and predicted risk of screening was calculated and plotted in quintiles using risk predictiveness curves that show the distribution of observed and predicted risks of completing screening. (Pepe et al., 2008, Lumley et al., 2002) Discrimination, or the ability to determine who will screen versus not, was measured by a bootstrap corrected C-statistic. (Moons et al., 2019) The explained variation was measured with an R^2^ statistic, an R^2^ of 0% means the model failed to explain variation, an R^2^ of 100% means the model explained all of the variation. (Royston, 2006). The calibration was measured by the Integrated Calibration Index (ICI), which assesses the difference between the model’s calibration and perfect calibration. (Austin and Steyerberg, 2019) Validation of the model was internal using bootstrapping. All analysis was initially conducted in STATA 17©, and then replicated in SAS (bootstrapping, C and R^2^) and R (ICI) by a KPNW analyst.

The first model was developed in a full dataset of patients who are due for CRC screening using individual level data from the EHR. The multilevel model used the same individual level dataset and incorporated multilevel data from databases at KPNW, and publicly available data. The statistical improvements in the model were then assessed when multilevel data was added. The model was validated internally using the preferred bootstrapping approach, which is a way to determine concordance and predict the fit of a model to a series of hypothetical datasets when other validation techniques are not available. (Steyerberg and Harrell, 2016) Bootstrapping is superior to separate training and testing of the data. (Austin and Steyerberg, 2017).

Data was first accessed for availability at each level of the SEM framework. Predictors were omitted from analysis due to missingness, and imputation was performed when possible. *BMI* had 9.0% of the values missing, imputation was performed by age group. Imputation was conducted in STATA, which uses the predictive mean matching imputation method. Language is not an imputable variable, so patients missing *language* (3.6%) were grouped with the most common category, English. Biological *sex* had 15 missing values (0.02%), these patients with missing data were removed from the analysis, as the models fit a population with no missing data.

## 3 Results

### 3.1 Individual level model (EHR data)

The full model included 98.97% of the population (n = 59,234) as 15 patients were missing *sex* and removed from the analysis. The screening outcome was completed for 36.1% of the population (n = 21,337), 90.7% were screened by FIT, and 14% were screened by colonoscopy (not mutually exclusive, patients may have been screened by more than one modality, data not shown). Less than 1% of patients were screened by Flexible Sigmoidoscopy, FIT DNA, or virtual colonoscopy. In the population, 6,041 (10.0%) discontinued KPNW insurance, and 369 (0.6%) died during the year of follow-up.

The variables in the full model included 21 individual characteristics (Table 1). Two variables, prostate screening, and mammogram screening were combined with sex. This leaves 19 remaining variables in the initial full model. The performance of the full model was adequate with a naive C-statistic of 0.724 and an R^2^ of 0.112. The step-down process was then used to simplify the model, based on Akaike’s information criteria (AIC) and the change in R^2^ (Steyerberg, 2019). First, the AIC was determined for each variable, variables were ranked from lowest to highest, and the least contributory variables were removed one by one in AIC order until the model R^2^ dropped to no lower than 0.1105 (99%). *Insurance group, language group, interpreter, BMI, inpatient visits,* were all removed from the model while retaining 99% of the predictive value.

**Table 1:** Screened and Unscreened Participant Characteristics (n = 59,234).

**Individual Characteristic**	**Without screeningn = 38,516**		**With screeningn = 21,704**	
	n	(%)	n	(%)
**Age in years (mean (SE))**				
50–55	10,779	(28.0)	6870	(31.7)
55–60	9443	(24.5)	4340	(20.0)
60–65	7909	(20.5)	4205	(19.4)
65–70	5667	(14.7)	3322	(15.3)
70+	4074	(10.6)	2640	(12.2)
**Ethnicity (Hispanic)**	1989	(5.2)	1261	(5.8)
**Insurance**				
Medicaid	1855	(4.8)	758	(3.5)
Medicare	9520	(24.7)	5958	(27.5)
Commercial	27,141	(70.5)	14,988	(69.1)
**Interpreter needed**	1835	(4.8)	1053	(4.9)
**Language (English)**				
English	36,358	(94.4)	20,553	(94.7)
Spanish	915	(2.4)	539	(2.5)
Other	1243	(3.2)	612	(2.8)
**Race (White)**				
White	29,121	(75.6)	17,444	(80.4)
Asian	1869	(4.9)	1299	(6.0)
Black	1664	(4.3)	920	(4.2)
Hawaiian/Pacific Islander	320	(0.8)	176	(0.8)
American Indian	369	(1.0)	198	(0.9)
Other	234	(0.6)	107	(0.5)
Unknown	4939	(12.8)	1560	(7.2)
**Sexand other cancer screening**				
Male + prostate screening	4622	(12.0)	3069	(14.1)
Male + no prostate screening	13,998	(36.3)	6994	(32.2)
Female + mammogram	14,523	(37.7)	10,535	(48.5)
Female no mammogram	5364	(13.9)	1100	(5.1)
BMI*				
Underweight	292	(0.8)	127	(0.6)
Normal	6772	(17.6)	4286	(19.7)
Overweight	15,562	(40.4)	7827	(36.1)
Obese	15,890	(41.3)	9464	(43.6)
Dental coverage and visits				
**Dentalplan andvisit in prior year**	6518	(16.9)	5608	(25.8)
No dental plan	24,414	(63.4)	12,525	(57.7
Plan, no visit	7584	(19.7)	3571	(16.5)
Plan, had visit	6518	(16.9)	5608	(25.8)
**Out-patient Visits**				
0	10,103	(26.2)	3427	(15.8)
1	5632	(14.6)	3124	(14.4)
2	4428	(11.5)	2691	(12.4)
3	3332	(8.7)	2179	(10.0)
4	2525	(6.6)	1612	(7.4)
5	2038	(5.3)	1313	(6.0)
6	1534	(4.0)	1074	(4.9)
7	1287	(3.3)	911	(4.2)
8	1039	(2.7)	757	(3.5)
9	878	(2.3)	584	(2.7)
10+	5720	(14.9)	4032	(18.6)
**In-patient visits in prior year > 0**	1672	(4.3)	792	(3.6)
**Missed a visit in past year**				
0	30,597	(79.4)	17,397	(80.2)
1	4706	(12.2)	2818	(13.0)
2+	3213	(8.3)	1489	(6.9)
**Membership > 5 years**	19,137	(49.7)	13,371	(61.6)
**Membership < 2 years**	7742	(20.1)	3133	(14.4)
**Patient portal enrollment (****kp.org****)**	26,527	(68.9)	17,615	(81.2)
**Comorbidity Score > 0**				
0	28,586	(74.2)	15,741	(72.5)
1	5143	(13.4)	3324	(15.3)
2+	4787	(12.4)	2639	(12.2)
**Tobacco or other substance**	12,286	(31.9)	7248	(33.4)
**Flu shot in prior year**	17,230	(44.7)	13,099	(60.4)
**Prior Screening**				
No prior screening	26,920	(69.9)	8756	(40.3)
Prior FIT	10,570	(27.4)	11,886	(54.8)
Prior Colonoscopy	1026	(2.7)	1062	(4.9)
*Imputed				

The 14 retained characteristics include *prior CRC screening and preference, age group, prior preventive screening and sex,* enrollment in KP.org (*patient portal*)*, dental membership and visits, number of outpatient visits, number of missed appointments, prior influenza vaccination, Charlson comorbidity score, race, Hispanic ethnicity, membership for 5 years or more, membership for less than 2 years,* and *substance abuse* (Table 2.)*.*

**Table 2:** Individual Level Data Model* (n = 59,234).

**Characteristic**	**Odds ratio**	**(95% CI)**		***p* value**
**Age in years (mean (SE))**				
50–54	ref			
55–59	0.47	(0.45	, 0.50)	0.00
60–64	0.53	(0.50	, 0.56)	0.00
65–69	0.53	(0.50	, 0.57)	0.00
70–75	0.52	(0.49	, 0.56)	0.00
**Ethnicity (Hispanic)**	1.43	(1.30	, 1.58)	0.00
**Race**				
White	ref			
Asian	1.20	(1.11	, 1.30)	0.00
Black	1.05	(0.94	, 1.18)	0.36
Hawaiian/Pacific Islander	0.92	(0.75	, 1.12)	0.40
American Indian	0.86	(0.71	, 1.03)	0.10
Other	0.77	(0.60	, 1.00)	0.05
Unknown	0.72	(0.66	, 0.78)	0.00
**Sex and other cancer screening**				
Male + prostate screening	ref			
Male + no prostate screening	1.02	(0.96	, 1.08)	0.52
Female + mammogram	1.04	(0.98	, 1.10)	0.16
Female no mammogram	0.58	(0.53	, 0.64)	0.00
Dental coverage and visits				
**Dentalplan andvisit in prior year**				
No dental plan	ref			
Plan, no visit	0.89	(0.84	, 0.93)	0.00
Plan, had a visit	1.31	(1.25	, 1.37)	0.00
**Prior Screening**				
No prior screening	ref			
Prior FIT	3.81	(3.66	, 3.98)	0.00
Prior Colonoscopy	3.23	(2.94	, 3.56)	0.00
**Out-patient Visits**				
0	ref			
1	1.37	(1.29	, 1.46)	0.00
2	1.45	(1.35	, 1.55)	0.00
3	1.54	(1.43	, 1.66)	0.00
4	1.52	(1.40	, 1.66)	0.00
5	1.53	(1.40	, 1.68)	0.00
6	1.68	(1.52	, 1.86)	0.00
7	1.69	(1.52	, 1.88)	0.00
8	1.74	(1.55	, 1.95)	0.00
9	1.57	(1.38	, 1.78)	0.00
10+	1.83	(1.70	, 1.97)	0.00
**Missed a visit in past year**				
0	ref			
1	0.85	(0.81	, 0.90)	0.00
2+	0.64	(0.59	, 0.69)	0.00
**Membership > 5 years**	1.18	(1.13	, 1.23)	0.00
**Membership < 2 years**	0.79	(0.75	, 0.84)	0.00
**Patient portal enrollment**	1.40	(1.34	, 1.47)	0.00
**Comorbidity Score**				
0	ref			
1	0.90	(0.86	, 0.95)	0.00
2+	0.70	(0.66	, 0.75)	0.00
**Flu shot in prior year**	1.30	(1.25	, 1.35)	0.00
**Tobacco or other substance**	0.88	(0.84	, 0.92)	0.00
*Intercept 0.298 (0.271, 0.326)				

**Fig. 2:**
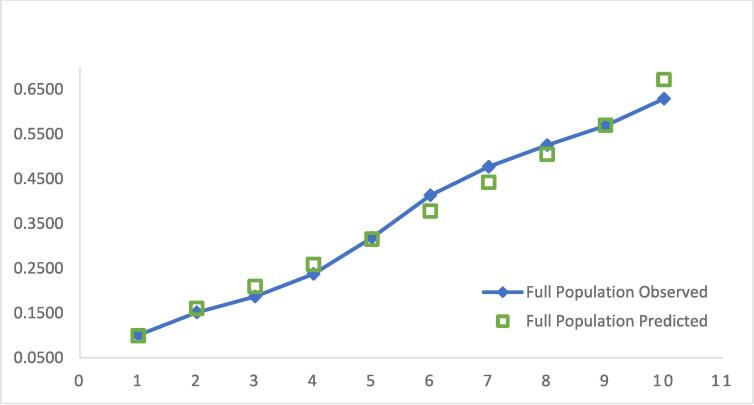
Individual Level Model Calibration Plot.

The performance measures used for evaluation were bootstrapped C and R^2^ and calibration. The model was also validated internally using bootstrapping (500 bootstraps) as described, which showed adequate performance with a bootstrap corrected C-statistic of 0.722 (Table 3). The calibration was also determined by plotting the observed and predicted risks of the reduced model by quintiles of predicted risk (Fig. 2). Calibration was also determined by calculating the ICI, which shows excellent calibration (0.013). If the observed and predicted values agreed perfectly, the ICI would be 0.0. Consequently, the model is only miscalibrated by an average probability of 0.01. The calibration of the observed and predicted risk appears to be sufficient, with close calibration between observed and predicted risk at all levels.

**Table 3:** Performance Characteristics of Individual and Multilevel Models.

	**Full Population**	
**Statistic**	**Individual Level**	**+ Multilevel Data**
**Number of observations**	59,234(0.02% missing data, n = 15)	58,040(2% missing data, n = 1194)
**C-statistic**	0.72	0.72
**Bootstrap-corrected C-statistic**	0.72	0.72
**R^2^ (95% CI)**	0.11	0.11
**Integrated calibration index (ICI)**	0.01	0.01
R^2^ statistic, represents the model’s predictive ability, the agreement between an individual’s predicted and observed risk of the event; ICI statistic represents the model's weighted difference between observed and predicted probabilities

### 3.2 Multilevel model

The multilevel model incorporates data outside of the individual level based on the SEM Framework. The full population model was fit for the multilevel data in the same way it was fit for the individual level model described above. The starting population was identical to the individual model. Patients were also removed from the full model due to missing data (n = 1,194). Missing data included patients who were missing *sex* (n = 15), interpersonal level data (i.e., *address changes* n = 186), provider level data (n = 415), clinic level data (n = 159), and miscellaneous information at the county level (i.e., *facilities* was missing for n = 834 patients). The remaining sample size in the full model is 98.0% of the full population (n = 58,040). The screening outcome was completed for 36.3% of the population in the model (n = 21,068).

The 57 variables included in the full model include the 19 individual characteristics described above, 5 interpersonal characteristics, 3 organizational characteristics, 29 community characteristics, and 1 policy level characteristic. Collinearity was assessed among the variables in the multilevel model. At the organizational level, the *patient to provider ratio* variable was redundant with clinic size and *clinic location (city)* was redundant with *primary care clinic;* the program removed *patient to provider ratio* and *clinic location* from the model*.*

The performance of the full model was adequate with a naive C-statistic of 0.725 and an R^2^ of 0.113. The model used 110 degrees of freedom. The step-down process was then used to simplify the model, based on AIC and the change in R^2 28^, using the same process as in the initial model.

The multilevel model retained 23 variables after stepdown (Table 4). All variables from the reduced, individual level model were included, as well as *inpatient visits* and *BMI*. Variables retained from the interpersonal level were *address changes* and *provider gender match.* From the organizational level, *wait time, clinic size,* and *primary clinic* were retained. From the community level, *facilities* (number of healthcare facilities > 15/100 K residents) and *median family income* were retained. From the policy level, the only variable (*STATE*) was not retained.

**Table 4:** Multilevel Data Model* (n = 58,040).

**Characteristic**	**Odds ratio**	**(95% CI)**		***p* value**
**Individual Level Characteristics**				
**Age in years**				
50–54	ref			
55–59	0.47	(0.44	, 0.49)	0.00
60–64	0.53	(0.50	, 0.56)	0.00
65–69	0.53	(0.50	, 0.57)	0.00
70–75	0.52	(0.48	, 0.55)	0.00
**Ethnicity (Hispanic)**	1.40	(1.26	, 1.54)	0.00
**Race**				
White	ref			
Asian	1.19	(1.09	, 1.29)	0.00
Black	1.09	(0.97	, 1.23)	0.13
Hawaiian/Pacific Islander	0.91	(0.74	, 1.11)	0.34
American Indian	0.85	(0.70	, 1.02)	0.09
Other	0.80	(0.62	, 1.04)	0.10
Unknown	0.73	(0.67	, 0.80)	0.00
**Sexand other cancer screening**				
Male + prostate screening	ref			
Male + no prostate screening	1.02	(0.96	, 1.09)	0.50
Female + mammogram	1.04	(0.98	, 1.10)	0.16
Female no mammogram	0.59	(0.54	, 0.65)	0.00
**BMI**				
Underweight	ref			
Normal	1.25	(0.99	, 1.57)	0.60
Overweight	1.22	(0.97	, 1.53)	0.94
Obese	1.15	(0.92	, 1.45)	0.23
**Dentalplan andvisit in prior year**				
No dental plan	ref			
Plan, no visit	0.90	(0.85	, 0.94)	0.00
Plan, had a visit	1.31	(1.25	, 1.37)	0.00
**Out-patient Visits**				
0	ref			
1	1.37	(1.29	, 1.46)	0.00
2	1.44	(1.35	, 1.55)	0.00
3	0.54	(1.43	, 1.66)	0.00
4	1.53	(1.41	, 1.67)	0.00
5	1.53	(1.40	, 1.68)	0.00
6	1.67	(1.51	, 1.85)	0.00
7	1.70	(1.53	, 1.89)	0.00
8	1.74	(1.55	, 1.96)	0.00
9	1.57	(1.39	, 1.79)	0.00
10+	1.88	(1.75	, 2.02)	0.00
**In-patient visits in prior year > 0**	0.81	(0.73	, 0.89)	0.00
**Missed a visit in past year**				
0	ref			
1	0.86	(0.81	, 0.91)	0.00
2+	0.67	(0.62	, 0.72)	0.00
**Membership > 5 years**	1.17	(1.12	, 1.23)	0.00
**Membership < 2 years**	0.79	(0.75	, 0.84)	0.00
**Patient portal enrollment**	1.40	(1.34	, 1.47)	0.00
**Comorbidity Score**				
0	ref			
1	0.92	(0.87	, 0.97)	0.00
2+	0.74	(0.69	, 0.79)	0.00
**Flu shot in prior year**	1.30	(1.25	, 1.35)	0.00
**Tobacco or other substance**	0.88	(0.85	, 0.92)	0.00
**Prior Screening**				
No prior screening	ref			
Prior FIT	3.82	(3.66	, 3.99)	0.00
Prior Colonoscopy	3.24	(2.94	, 3.57)	0.00
**Multilevel Data**				
**Address changes**				
0	ref			
1	1.00	(0.95	, 1.05)	0.85
2+	0.90	(0.84	, 0.96)	0.00
**Provider gender match**	1.07	(1.03	, 1.12)	0.00
**Clinic assignment**				
0	ref			
1	0.43	(0.27	, 0.68)	0.00
2	0.50	(0.34	, 0.74)	0.00
3	0.74	(0.35	, 1.57)	0.43
4	0.83	(0.47	, 1.47)	0.53
5	1.01	(0.57	, 1.81)	0.96
6	0.43	(0.27	, 0.69)	0.00
7	0.98	(0.55	, 1.74)	0.94
8	0.50	(0.34	, 0.74)	0.00
9	0.48	(0.25	, 0.95)	0.03
10	1.05	(0.59	, 1.87)	0.86
11	1.01	(0.58	, 1.79)	0.96
12	0.90	(0.75	, 1.08)	0.27
13	0.46	(0.29	, 0.72)	0.00
14	0.48	(0.33	, 0.72)	0.00
15	0.92	(0.52	, 1.65)	0.79
16	0.48	(0.30	, 0.75)	0.00
17	0.50	(0.32	, 0.78)	0.00
18	1.09	(0.61	, 1.94)	0.77
**Wait time**				
<=30 days	ref			
31–45 days	0.98	(0.59	, 1.62)	0.94
45 + days	1.04	(0.60	, 1.80)	0.89
**Clinic size > 30,000**	1.94	(1.37	, 2.74)	0.00
**Median household income**				
<45 K	ref			
45–85 K	1.05	(0.95	, 1.16)	0.31
85–140 K	1.22	(1.01	, 1.24)	0.03
140 K+	1.14	(1.00	, 1.30)	0.05
**Number of healthcare facilities (>15/100 K)**	1.09	(1.02	, 1.16)	0.01
*Intercept 0.235 (CI 0.125, 0.443)				

**Fig. 3:**
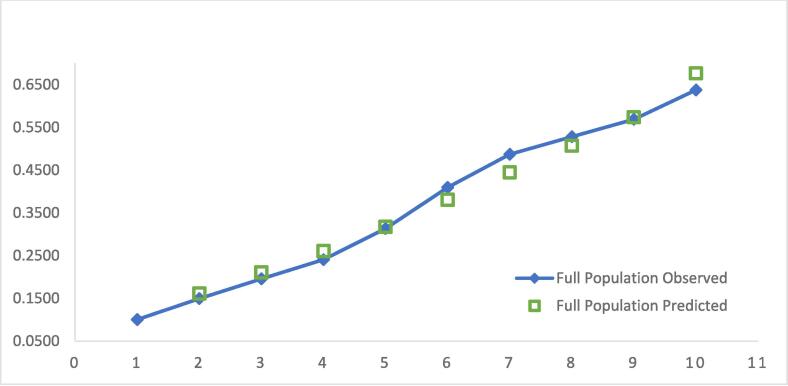
Multilevel Model Calibration Plot.

The final reduced model R^2^ was 0.1119 and had 70 degrees of freedom, the reduced C-statistic was 0.7242, and the ICI is 0.0130 (Table 3). Bootstrapping and calibration were used to evaluate the performance of the model. The model was validated internally using bootstrapping (500 bootstraps), which showed adequate performance with a bootstrap corrected C-statistic of 0.7218. The calibration was also determined by plotting the observed and predicted risk of the reduced model (Fig. 3).

### 3.3 Model comparison

The overall screening population (eligible for this analysis) was approximately 9.8% (59,249/617,073) of the overall age eligible population of KPNW (Appendix Table A1). Compared to the overall population of KPNW, a greater proportion of the population identified as due for screening, female, Native American or American Indian, White, and enrolled in Medicare. There are fewer patients in the screening population who are Asian, Black, Hawaiian or Pacific Islander, patients that list “Unknown” race, Hispanic ethnicity, and have Medicaid insurance. The population that completed screening is 52.7% female; 6.1% Asian; 2.8% Black; less than1% Hawaiian or Pacific Islander, Native America or American Indian, or identify as “other” race;7.3% have “unknown” race; 81.8% White; 5.9% Hispanic; and 27.9% are on Medicare; and 3.4% are on Medicaid (Table 1.).

The individual model intercept is 0.298 (95% CI 0.271, 0.326), and the multilevel model intercept is 0.235 (95% CI 0.125, 0.443). The clinical utility of the models could be used in mapping predicted probabilities of screening. To apply clinical utility, we have presented points for the variables in each model (Appendix A2.).

A comparison analysis was conducted to ensure that the model was not only applicable to the prevalent Northwest demographic population (insured and White patients). Subpopulations considered for a comparison analysis were the Medicaid and non-White groups. The Medicaid patient subgroup (n = 2613) was determined to be too small to handle the multilevel analysis. While we would have ideally been able to apply the model to individual subgroups of patients historically underserved by the medical system, no single group had enough cases for individual analysis. Therefore, the *non-White* group (n = 13,655) was the subpopulation for comparative analysis. To assess the risk of algorithmic bias in the models, they were fit in a non-White subpopulation.

In the individual model for the subgroup analysis, the model was fit for 99.94% of the subpopulation (n = 12,676), 8 patients were missing sex and eliminated from the model. The screening outcome was completed for 31.0% of the 50–75 aged population (n = 3,933). The individual model in the subpopulation was slightly improved from the full population with an R^2^ of 0.1364, C-statistic of 0.7490, and ICI of 0.0183 (Appendix Table A2). The multilevel model was fit for 96.05% of the population (n = 12,184) in the subpopulation. Patients were missing data (n = 500) as described above in the full multilevel model, including missing provider, clinic, and community level data. The screening outcome was completed for 31.5% of the population (n = 3,838). The subpopulation multilevel model was slightly improved from the full population with an R^2^ of 0.1369, and the C-statistic of 0.7484, and the ICI is 0.0206.

## 4 Discussion

Predictive analytics and multilevel data are increasingly used in population health management and offer clinical decision support at the point of care. The use of predictive analytics can allow systems to personalize care based on an individual’s risk of certain events. (Parikh et al., 2016) Our findings show that an individual reduced model using 14 variables commonly available in EHR databases was able to adequately predict CRC screening to be used in clinical care to optimize the delivery of CRC promotion efforts. The multilevel reduced model, which added components at the individual, interpersonal, organizational, and community levels (and used 23 variables), only modestly improved model performance. The bootstrap-corrected C-statistics are identical; the R^2^ statistic is only 1% higher, and the ICI is 3% better on a relative scale for the multi-level model.

No other models that test the likelihood of obtaining screening for CRC were identified in the peer-reviewed literature. However, past literature has found that risk prediction is useful in tailoring screening programs to inform patients of the risk of developing cancers (Saya et al., 2020). Some models have used a few available multilevel variables, but no risk prediction models have focused on the performance improvement after the addition of multilevel data. The lack of information about adding multilevel data could be attributed to limitations in the availability of these data, and the lack of contributions to the model, as we found.

There is potential clinical use for either the individual or multilevel model. There is a pragmatic use of the reduced individual model that is the most simplistic way to identify patients’ likelihood of screening. In this sense, the individual model could be used to target patients for interventions aimed at increasing indicated screening. In addition, the parsimony of the individual model reduces the effort and potential resources needed to acquire and maintain multilevel data as well as the generalizability outside KPNW or other closed systems of care (e.g., VA). Our model holds promise to serve as a tool to efficiently identify patients’ likelihood of screening, so that screening promotion efforts could be directed to those most likely to benefit. Both the individual model and multilevel models are sufficient in discrimination (C > 0.6) (Steyerberg et al., 2010).

The literature has addressed the benefits of looking at multiple levels of data and the groups to which people belong. The literature shows that the different levels are linked or interconnected and that levels can be synergistic (Rousseau, 1985, Weiner et al., 2012, Taplin et al., 2012) Nevertheless, the multilevel data did not have the expected impact on our model. This may be because the multilevel variables’ influence on screening may be captured by already included individual level data that acts as a proxy for multilevel data. Or the KPNW patient population may be too homogenous to capture community and policy level differences. Multilevel data that may be beneficial to models is not always available in EHR and administrative databases. For example, income, education, marital status and physician recommendation are known predictors of screening yet were unavailable in the data (Beydoun and Beydoun, 2008, Guessous et al., 2010, Zimmerman et al., 2006, Laiyemo et al., 2014, Seeff et al., 2004). Further, multilevel data may be less reliable than individual level data.

Applicability was assessed for a non-White population. Both models showed improved performance after application to the non-White patients, reassuring concerns of applicability when applied diverse populations. Further, the non-White population, was chosen partly because they have greater variation in screening rates. The screening outcome was present in 36.1% of the full population and only 31.2% of the non-White subpopulation. Rates of CRC (adenoma prevalence) have been found to be higher in Black patients but determined to be predominately due to lower rates of screening. (Rutter et al., 2021) The non-White population (n = 13,655) provided an adequate subgroup for the application of the large model. Both the individual and multilevel models performed better statistically in the non-White population than in the full population, as the R^2^ and C-statistics in both models were both higher. The health system should use the non-White version specific to that population, as the model calibration is better in the sub-population model.

There are several limitations in this study. Ideally, a model would be created using data from multiple health systems with diverse geographical and patient populations, for a greater understanding of applicability to subpopulations and generalizability. While internal validity was determined through bootstrapping, transferability will have to be tested through external validation. The model would need to be redeveloped in the population where it is to be used, as there may be different dependencies in individual and multi-level data due to factors like culture. This model was developed in an integrated delivery and financial system (KPNW) in a single geographic region (Pacific Northwest). The geographical (community level) and organizational variation limit the impact of multilevel data, where it could be more valuable in a wider and more diverse population.

There are a variety of ways this project could expand to future research. A series of projects could externally validate the model. Externally validating the model in community clinics, other integrated systems, other geographical areas, or in a combination of environments could determine a more broad-based utility and generalizability of the model. The multilevel data could improve the model in other environments. Other multilevel data that was not included due to lack of distribution, unavailability, or missingness could improve a model in another setting. This model could also be used to prioritize interventions to increase CRC screening.

**Disclosure of Funding and Conflicts of Interest**

Analysis time for this project was donated by the KPNW Center for Health Research to create a student dataset for analysis. Dr. Petrik’s time during manuscript preparation was supported by grant number K12HS026370 from the Agency for Healthcare Research and Quality. The content is solely the responsibility of the authors and does not necessarily represent the official views of the Agency for Healthcare Research and Quality. No organization outside of the committee had any role in the design, implementation, interpretation, and reporting of this project.

From 2020–present, Dr. Coronado has served as a Scientific Advisor on contracts through the Center for Health Research for Exact Sciences and Guardant Health. Dr. Coronado is also the PI, and Dr. Mummadi a co-Investigator on a contract through the Center for Health Research funded by Guardant Health that is assessing adherence to a commercially available blood test for colorectal cancer. All other authors declare no potential conflicts of interest.

## Declaration of Competing Interest

The authors declare that they have no known competing financial interests or personal relationships that could have appeared to influence the work reported in this paper.

## Figures and Tables

**Table A1:** Screening Population Comparison.

		Total KPNW Population*	**CRC Screening Population**	**Screened Population**
	Total	617,073	59,249	21,337
**Gender**	Female	51.8%	52.4%	53.7%
**Race**	Asian	6.1%	5.4%	6.1%
	Black	3.4%	2.7%	2.8%
	Hawaiian/Pacific Islander	1.0%	0.8%	0.8%
	Native American/American Indian	0.8%	1.0%	0.9%
	Other	0.6%	0.6%	0.5%
	Unknown	22.7%	11.0%	7.3%
	White	65.5%	78.6%	81.6%
**Ethnicity**	Hispanic	8.4%	5.5%	5.9%
**Insurance**	Medicare	18.4%	26.7%	28.3%
	Medicaid	10.7%	4.2%	3.4%
*Results as of 2/28/21				

**Table A2:** Performance statistics for all prediction models.

	**Full Population**		**Subpopulation (Non-White)**	
**Statistic**	**Individual Level**	**+ Multilevel Data**	**Individual Level**	**+Multilevel Data**
**Number of observations**	59,234(0.02% missing data, n = 15)	58,040(2% missing data, n = 1194)	12,676 (0.06% missing data, n = 8)	12,184 (3.94% missing data, n = 500)
**C-statistic**	0.7232	0.7242	0.7501	0.7505
**Bootstrap-corrected C-statistic**	0.7220	0.7218	0.7457	0.7384
**R^2^ (95% CI)**	0.1108	0.1119	0.1364	0.1369
**Integrated calibration index (ICI)**	0.0134	0.0130	0.0183	0.0206

R^2^ statistic, represents the model’s predictive ability, the agreement between an individual’s predicted and observed risk of the event; ICI statistic represents the model's weighted difference between observed and predicted probabilities.

**Table A3:** Model Points per Predictive Characteristic.

	**Individual Level Model**	**Multilevel Model**
Points per unit of linear predictor	74.68659	74.57246
Linear predictor units per point	0.01338928	0.01340978
Intercept	0.298	0.235
**Characteristic**	**Points**	**Points**
Age in years (mean (SE))		
50–54	56	57
55–59	0	0
60–64	9	10
65–69	9	10
70–75	8	8
Ethnicity (Hispanic)	27	25
Race (White)		
White	25	23
Asian	38	36
Black	28	30
Hawaiian/Pacific Islander	18	16
American Indian	13	11
Other	5	7
Unknown	0	0
Sex		
Male + prostate screening	40	39
Male + no prostate screening	42	41
Female + mammogram	43	42
Female no mammogram	0	0
BMI		
Underweight	–	0
Normal	–	17
Overweight	–	15
Obese	–	10
Dental visit in prior year		
No dental plan	9	8
Plan, no visit	0	0
Visit	29	28
Out-patient Visits		
0	0	0
1	24	24
2	28	27
3	32	32
4	31	32
5	32	32
6	39	38
7	39	39
8	41	41
9	34	34
10+	45	47
In-patient visits in prior year > 0	–	16
Missed a visit in past year		
0	33	30
1	22	19
2+	0	0
Membership > 5 years	12	12
Membership > 2 years	17	17
Patient portal enrollment	25	25
Comorbidity Score		
0	27	23
1	19	16
2+	0	0
Flu shot in prior year	19	19
Tobacco or other substance	10	9
Prior Screening		
No prior screening	0	0
Prior FIT	100	100
Prior Colonoscopy	88	88
**Multilevel Data**		
Address changes		
0	–	8
1	–	8
2+	–	0
Provider gender match	–	5
Clinic assignment		
0	–	63
1	–	0
2	–	11
3	–	41
4	–	49
5	–	64
6	–	1
7	–	61
8	–	12
9	–	9
10	–	67
11	–	64
12	–	55
13	–	5
14	–	9
15	–	57
16	–	8
17	–	11
18	–	69
Wait time		
<=30 days	–	1
31–45 days	–	0
45 + days	–	4
Clinic size > 30,000	–	49
Median household income		
<45 K	–	0
45–85 K	–	4
85–140 K	–	9
140 K+	–	10
Number of healthcare facilities (<15/100 K)	–	6

## Data Availability

Data will be made available on request.

## Appendix

Table A1.

Table A2.

Table A3.
